# Posterior decompression and occipitocervical fixation followed by intraoperative vertebroplasty for metastatic involvement of the axis

**DOI:** 10.1186/s12891-018-1928-7

**Published:** 2018-01-11

**Authors:** Xinjie Wu, Mingsheng Tan, Yingna Qi, Ping Yi, Feng Yang, Xiangsheng Tang, Qingying Hao

**Affiliations:** 1 Department of Spinal Surgery, China-Japan Friendship Hospital, Beijing, 100029 People’s Republic of China; 2 Graduate School of Peking Union Medical College, Beijing, 100730 People’s Republic of China; 3 Graduate School of Beijing University of Chinese Medicine, Beijing, 100029 People’s Republic of China

**Keywords:** Vertebroplasty, Occipitocervical fixation, Axis, Metastasis

## Abstract

**Background:**

Metastases to the upper cervical spine were rarely reported in the literature. However, metastases to this area may cause spinal instability and cord compression, which in turn can result in respiratory failure and neurological dysfunction. The present study investigated the efficacy and safety of posterior decompression and occipitocervical fixation followed by intraoperative vertebroplasty for this disease.

**Methods:**

This was a retrospective study that included 10 patients with metastatic involvement of the axis from March 2002 to May 2014. All cases presented with occipitocervical pain: 5 patients with compressive myelopathy and 6 patients with radiculopathy. Japanese Orthopedic Association (JOA) scores and Visual Analogue Scale (VAS) were used to evaluate the improvement of neurological function and pain intensity, respectively.

**Results:**

All patients underwent posterior decompression and occipitocervical fixation followed by intraoperative vertebroplasty. The VAS scores and JOA scores both improved postoperatively, from 8.2 ± 0.4 to 2.3 ± 0.2 and from 10.1 ± 2.2 to 14.2 ± 2.9, respectively. Additionally, the improvement rate of JOA was 52.4 ± 1.8%. The mean overall survival was 12.8 months. The median survival time was 7 months. The 6-month and 12-month survival rates were 70% and 40%, respectively. The mean duration of operation was 182 min and blood loss was 450 mL. The mean volume of bone cement injected was 4.0 mL. The cement extravasation was observed in only 1 patient without clinical symptoms. One patient developed tumour recurrence and died 1 month later.

**Conclusions:**

Posterior decompression and occipitocervical fixation followed by intraoperative vertebroplasty was a safe and valuable palliative method with relatively less invasion to treat metastatic involvement of the axis.

## Background

The spine ranks first in the list of cancer metastasis in the skeletal system. Approximately 8-20% of metastases occur in the cervical spine [1–3]. However, metastases to the upper cervical spine were rarely reported in the literature [4, 5]. Due to the importance of this area, metastatic involvement of the axis may cause spinal instability and cord compression, which in turn can result in respiratory failure and neurological dysfunction. For advanced patients, the aim of surgical intervention was not to cure the disease but to relieve the pain and spinal cord compression, reconstruct spinal stability, and improve the quality of remaining life. The treatments included external immobilization, conventional surgical resection and percutaneous cement augmentation. However, conventional surgical treatment itself had risks of mixed infection, speech change, tongue oedema, cerebrospinal fluid leakage, etc., due to poor clinical conditions, comorbidities, and its transoral approach, while percutaneous vertebroplasty in this area was challenging [6, 7].

Therefore, combining intraoperative vertebroplasty and posterior decompression and occipitocervical fixation may be advisable, which is less invasive and could provide strong stability. To our knowledge, palliative surgical treatment of such disease has rarely been reported. This palliative method was used to treat 10 patients with metastatic involvement of the axis in our study and produced desirable outcomes.

## Methods

### Patients

Between March 2002 and May 2014, 10 patients (7 males, 3 females) with metastatic involvement of C2 who underwent surgery were retrospectively analysed. The mean age was 68.9 years, ranging from 49 to 82. In this study, all cases presented with occipitocervical pain: 5 patients with compressive myelopathy, and 6 patients with radiculopathy. The clinical data of the patients are displayed in Table 1.

**Table 1:** Clinical data of patients

Parameters	n (total 10 patients)
Average age	68.9
Sex ratio (male/female)	7/3
Primary tumor	
Lung	4
Breast	2
Prostate	2
Kidney	1
Unknown	1
Clinical symptom	
Occipitocervical pain	10
Compressive myelopathy	5
Radiculopathy	6
Complication	
Cement extravasation	1
Tumor recurrence	1

### Surgical indication and contraindication

The indications for surgery were intractable pain resistant to nonsurgical treatment, instability, progressive neurologic dysfunction, and expected survival time>3 months. The contraindication included intolerance to surgery; severe heart, lung, liver or kidney diseases; and extensively widespread metastasis. Four patients were excluded from the study due to short life expectancy or poor conditions. Preoperatively, the Modified Tokuhashi Scoring System [8] was used to predict the survival period for the patients. Four patients had a score of 0-8 (life expectancy<6 months), while 6 patients had a score between 9 and 11 (life expectancy>6 months). Furthermore, the Spinal Instability Neoplastic Score (SINS) [9] was employed to determine spinal instability. Five patients had a SINS of 7-12 (indicating impending instability) and 5 patients had a SINS of 13-18 (indicating an unstable spine).

### Surgery preparation

All 10 patients underwent meticulous oncological examination preoperatively. The radiological data were evaluated using X-ray, CT scans and MRIs. In addition, radionuclide bone scanning was performed to further identify the location of the primary neoplasm and metastases. All procedures were performed by the same surgeon (M.S. Tan).

### Procedure

**Fig. 1:**
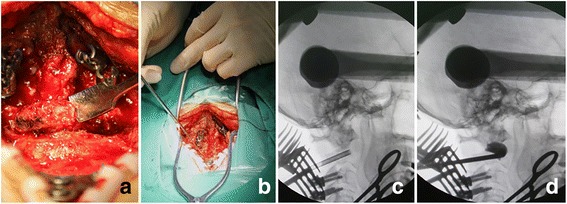
The surgical process. **a** Posterior decompression. **b** Intraoperative vertebroplasty via the transpedicular approach. **c** and **d** Intraoperative vertebroplasty under C-arm guidance

**Fig. 2:**
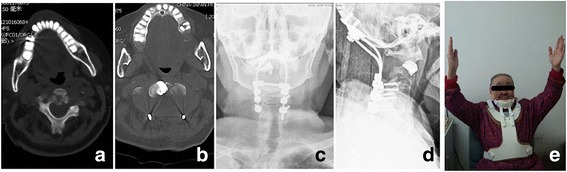
A 78-year-old female with a C2 metastatic tumour, presenting with occipitocervical pain and quadriplegia, was treated with intraoperative vertebroplasty combined with posterior decompression and occipitocervical fixation in 2012. **a** and **b** Preoperative and postoperative axial CT scans. **c** and **d** Postoperative anterior, posterior and lateral radiographs. **e** The muscle power of the patient returned to normal and pain was completely relieved

Neurological function was monitored by intraoperative somatosensory-evoked potentials. After endotracheal intubation and general anaesthesia, the patient was placed in the prone position with his or her head immobilized and slightly flexed by a Mayfield head holder. After disinfecting and routine draping, a standard midline incision was made to expose the posterior structure of the occipitocervical area. The laminar ligaments and muscles were dissected. Then, laminectomy and removing the superior and inferior facet joints and pedicle were performed using a high-speed burr and rongeur. The spinal cord, nerve root and vertebral artery were protected carefully during procedures. Transpedicular vertebroplasty with polymethylmethacrylate (PMMA) was performed during surgery using a C-arm image intensifier (Fig. 1). Intraoperative vertebroplasty was accurate and visual to inject bone cement. The leakage of bone cement could be detected and cleared in time. The PMMA was injected at high viscosity. Under C-arm guidance, the occipital plate and lateral mass screws or pedicle screws were placed and connected with a pre-curved rod bilaterally (O-C5) (Fig. 2). After irrigation, the incision was closed in layers and a drainage tube was placed inside.

Postoperatively, all patients were transferred to the oncology department for further treatment, such as radiotherapy or chemotherapy, as decided by the medical oncologists.

### Evaluation indexes

Postoperative outcomes were obtained and evaluated both clinically and radiologically. The Japanese Orthopedic Association (JOA) score was used to assess the improvement of neurological function. The formula of improvement rate of JOA was as follows: postoperative improvement rate = (postoperative score – preoperative score) / (17 – preoperative score) × 100%. The pain intensity was compared before and after operation by the VAS score, which had a scale of 0-10. Moreover, the dose of painkillers before and after the intervention was also recorded. For the radiological follow-up, plain X-ray film and CT scans were performed to evaluate the status of internal fixators and cement leakage, and MRIs were used to assess tumour recurrence. In addition, overall patient survival was calculated.

### Statistical analysis

Paired *t* test was used to compare changes before and after the operation with parametric values. The t test was considered significant if the *P* value level was less than 0.05. Statistical analyses were performed using SPSS 20.0 software.

## Results

### Surgical result

The mean duration of operation was 182 min (range 120-255) and intraoperative blood loss ranged from 250 to 850 mL (mean 450). The mean volume of bone cement injected was 4.0 mL, ranging from 3.0 to 5.0 mL. The amount of bone cement injected was calculated by measuring the remaining amount in the syringe. The cement extravasation was observed in only 1 patient according to postoperative CT scans. However, the cement leakage event was not related to clinical symptoms.

None of the significant complications, i.e., vertebral artery (VA) or spinal cord injuries, occurred during the operation or the follow-up period. Surgical site infection was not seen in our study. One patient had a pulmonary infection that resolved with antibiotic therapy. Similarly, no hardware failure or neurological worsening occurred during the whole follow-up period. However, 1 patient developed tumour recurrence and died 1 month later.

### Functional result

All patients received various extents of pain relief, which were sustained to the final follow-up period. Symptoms of myelopathy were presented in 5 patients, improved in 4 cases and remained unchanged in only 1 patient. All 6 patients who presented with radiculopathy obtained complete relief. The VAS scores and JOA scores both demonstrated improvement postoperatively (Table 2). In addition, the improvement rate of JOA was 52.4% (range: 49.6% to 57.1%). Due to the advanced stage of the tumours, all patients were prescribed oral morphine to relieve pain. The average preoperative daily dose was 125 mg (range: 80-150 mg), which decreased to an average of 60 mg (range: 50-100 mg) (P<0.001). The surgery significantly reduced the daily use of painkillers.

**Table 2:** VAS and JOA outcome

*n* = 10	Pre-op	Final follow-up	*P* value
VAS	8 (1)	2 (1)	*P* < 0.001
JOA	10 (2)	14 (2)	*P* = 0.003

Note: All data were expressed in median (interquartile range)

### Overall survival

All patients were followed up, until they died, for an average of 12.8 (range: 3 to 29) months. The overall median survival time was 7 months. The 6-month and 12-month survival rates were 70% and 40%, respectively. Furthermore, the mean overall survival period was 6.8 months for prostate cancer, followed by lung (7.4 months), kidney (10 months), unknown (10 months), and breast (27.5 months).

## Discussion

Metastasis to the spine are commonly spread through the Batson plexus, a valveless vertebral venous complex. Additionally, the vertebral body is the most common site to be affected compared with the posterior elements [10]. In our study, all cases had an affected vertebral body of C2. The clinical picture of metastatic involvement of the upper cervical region ranges from localized pain to a variable extent of the neurological deficits and even death induced by spinal cord compression [11, 12]. Occipitocervical pain occurred in approximately 89% to 100% of patients, was not linked to daily activities and worsened in the night [13–15]. All patients in our study presented with occipitocervical pain. Furthermore, given the relatively larger sagittal diameter of the C1 and C2 level compared to the counterpart of the subaxial cervical spine, the neurological deficit was overt only in the later stages. Hence, the preoperative JOA score was 10.1 ± 2.2 in our study.

With the development of internal fixators and surgical techniques, more invasive approaches were recommended for patients with upper cervical metastases who met strict selective criteria. The aim of the surgery for upper cervical metastases was to decompress the spinal cord, reconstruct spinal stability and obtain pain relief. The main goal was improving the quality of the remaining life rather than cure the disease. Previous studies suggested that metastases to the cervical spine have a worse prognosis than metastases to other sites [16, 17]. However, a recent matched pair analysis showed that metastatic spinal cord compression had similar clinical outcomes to those of thoracic and lumbar spine metastasis in terms of postoperative survival, surgery-related complication, and pain outcome [18].

Total *en bloc* spondylectomy (TES) was widely used to avoid local recurrence of spinal metastases. However, TES also had adverse aspects, such as a longer operation time, massive blood loss and difficulty to perform in the upper cervical spine because of the distinct anatomy of the vertebral arteries and the complex bony architecture. In addition, the transoral approach to the operative field was deep and narrow and many complications may occur, such as mixed infection, speech change, tongue oedema, retropharyngeal haematoma or cerebrospinal fluid leakage [6]. Sakaura et al. [19] described that the recurrence rate was similar for palliative surgery compared with TES. In our study, we only observed one patient with recurrence.

Vertebroplasty or kyphoplasty was widely used on spinal metastases and had gained effective clinical results in patients with osteolytic spinal metastases. Recently, kyphoplasty was even applied in osteoblastic spinal metastases and caused a significant reduction of pain [20]. The mechanism of pain relief was thought to be that bone cement stopped vertebral micro- or macromovement and the destruction of pain receptors through exothermic reactions and chemical and toxic effects [21]. Percutaneous vertebroplasty (PVP) has a shorter operation period, minimal blood loss, rare intra-operative injuries and low cost [22]. The relief of pain was commensurate with an increase in activity, which in turn decreased the risk of concomitant infections and the need for further hospitalization. In our study, all patients experienced a certain extent improvement in occipitocervical pain and a decrease in VAS from 8.2 ± 0.4 to 2.3 ± 0.2. However, this method could not address the problem of spinal cord compression. Interestingly, several studies demonstrated that PMMA had an antitumoural effect, which may be due to the cytotoxicity, thermal effects and ischaemic effects [23, 24]. However, others proposed that the low recurrence rate was attributed to the short survival time rather than the antitumoural effect. Hence, the antitumoural effect needs further study. In addition, transoral PVP also had the demerits itself.

Therefore, posterior surgery combined with intraoperative vertebroplasty may be a feasible and less invasive method. Intraoperative vertebroplasty was accurate and visual to inject bone cement. In our study, cement extravasation was only detected in 1 case, but with no clinical significance (10%).This was overtly lower than PVP (51%) [25]. According to our experience, it was important that the cement injection was under high viscosity, with intraoperative fluoroscopy and preoperative evaluation of the integrity of the vertebral endplates and posterior wall of the vertebrae. Furthermore, laminectomy and removing the pedicles could provide a better way to perform vertebroplasty and the possibility of an immediate clearing of the leaked bone cement.

In our study, our patients had either impending instability or an unstable spine according to SINS, of which sensitivity and specificity was 96% and 80%, respectively [26]. If patients were in good clinical and stable oncological condition, extended posterior fixation (O-C5) was highly recommended [14]. Patients in our study were long-term bedridden and malnourished, with a mean age of 68.9 years. In addition, the upper cervical spine lacked the support of ribs, which was different from the thoracic spine. Thus, all these factors contributed to the decision to perform an extended posterior fixation. This could achieve the maximum and most long-lasting stability. The shorter posterior fixation might later become insufficient and increases the risk of reoperation. Due to the nature of palliative surgery, we do not ordinarily perform a bone graft. This was consistent with a previous study [27].

Lung, breast and prostate cancer were the 3 main tumours that metastasized to the spine [28, 29], which accounted for 80% in our study. The average survival time of our study was 12.8 months, which was within the range of previous studies, 3-18 months [2, 30]. Patients with primary tumours from lung and prostate had the worst prognosis and a relatively shorter survival time. This was consistent with previous studies [31]. It was worth noting that postoperative radiotherapy and chemotherapy were also important in terms of prolonging life and improving the quality of life. Treatment of spinal metastases is challenging and needs multidisciplinary collaboration.

There were several limitations. First, there was the nature of the retrospective study. Second, the number of patients included was relatively limited due to the rarity of this disease. Third, there was the absence of the control group. A larger controlled study is required to identify the efficacy of this method.

## Conclusion

Posterior decompression and occipitocervical fixation followed by intraoperative vertebroplasty was a safe and valuable palliative method with relatively less invasion to treat metastatic involvement of the axis.

## Abbreviations

PMMA: Polymethylmethacrylate
PVP: Percutaneous vertebroplasty
SINS: Spinal Instability Neoplastic Score
TES: Total en bloc spondylectomy
VA: Vertebral artery
